# Endovascular Therapy as an Emerging Paradigm for the Treatment of Popliteal Artery Thrombosis Following Total Knee Arthroplasty: A New Approach to Acute Limb Ischemia Management

**DOI:** 10.3400/avd.oa.24-00068

**Published:** 2025-02-05

**Authors:** Tammiraju Iragavarapu, Gurrala Kartheek Krishna, Subhendra Nath Sobhanadri, Aditya Kota, V. Venkata Sushma

**Affiliations:** 1 HOD of Cardiology, Alluri Sitaramaraju Academy of Medical Sciences, Eluru, Andhra Pradesh, India; 2 Department of General Medicine, East Point College of Medical Sciences and Research, Bengaluru, Karnataka, India; 3 Department of General Medicine, Alluri Sitaramaraju Academy of Medical Sciences, Eluru, Andhra Pradesh, India; 4 Department of Orthopedics, Alluri Sitaramaraju Academy of Medical Sciences, Eluru, Andhra Pradesh, India; 5 Department of Pathology, Alluri Sitaramaraju Academy of Medical Sciences, Eluru, Andhra Pradesh, India

**Keywords:** popliteal artery, thrombosis, arthroplasty, endovascular intervention, thrombectomy

## Abstract

**Objectives:** The objective of this research was to examine the occurrence, clinical features, treatment approaches, and results associated with a rare complication of thrombosis of the popliteal artery after total knee arthroplasty (TKA), leading to acute limb ischemia (ALI).

**Methods:** A retrospective study on 1020 TKA procedures spanning 5 years. Cases of ALI were identified through clinical evaluation and arterial Doppler studies. Peripheral angioplasty was done to recanalize the popliteal artery. Manifestations, complications, and management strategies were evaluated.

**Results:** Among the 1020 TKA cases, 5 cases of ALI were identified which accounts for 0.49% of all TKA cases. Female predominance and left-sided presentations are notable observations. Most patients presented within 8 hours of symptom onset with diverse complications ranging from foot drop to compartment syndrome. Except for 1 case, all patients recovered with thrombosuction and balloon dilatation.

**Conclusions:** A rare but potentially fatal complication of TKA is popliteal artery thrombosis leading to ALI so it becomes important for early recognition and intervention to mitigate the adverse outcomes. In our study, endovascular treatment has emerged as the preferred modality in terms of effective management and reducing complications and morbidity from surgical procedures.

## Introduction

Total knee arthroplasty (TKA) or total knee replacement (TKR) is a common orthopedic surgery that involves replacing the articular surfaces (femoral condyles and tibial plateau) of the knee joint with smooth metal and highly cross-linked polyethylene plastic. It is a commonly recommended procedure for older patients with end-stage osteoarthritis, a group who are already susceptible to complications such as peripheral arterial disease. Approximately 2% of TKA patients were observed to be having chronic lower extremity arterial insufficiency.^1)^ Typical post-TKA complications include infection, dislocation, fracture, deep venous thrombosis (DVT), and popliteal artery thrombosis. Popliteal artery thrombosis is a rare complication and occurs at an incidence of 0.03%–0.17%.^2)^ Popliteal artery occlusion following TKA has a high mortality of up to 7% and a risk of amputation of up to 42% if not promptly identified and managed. This highlights the critical need for a swift diagnosis and management to prevent potential limb loss or fatal consequences.^3)^

## Materials and Methods

**Figure 1:**
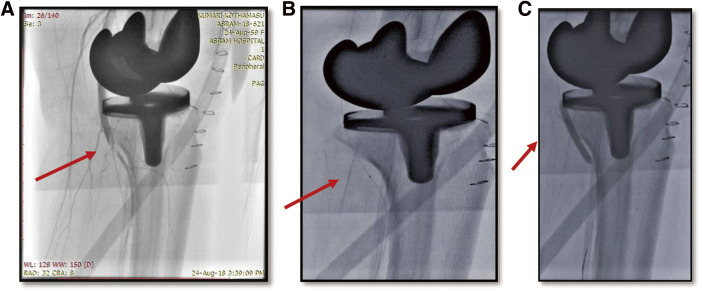
Fig. 1 (**A**) Image depicts the thrombotic occlusion of the popliteal artery (red arrow). (**B**) Thrombosuction using a thrombuster catheter (red arrow). (**C**) Balloon dilatation of the popliteal artery (red arrow).

**Figure 2:**
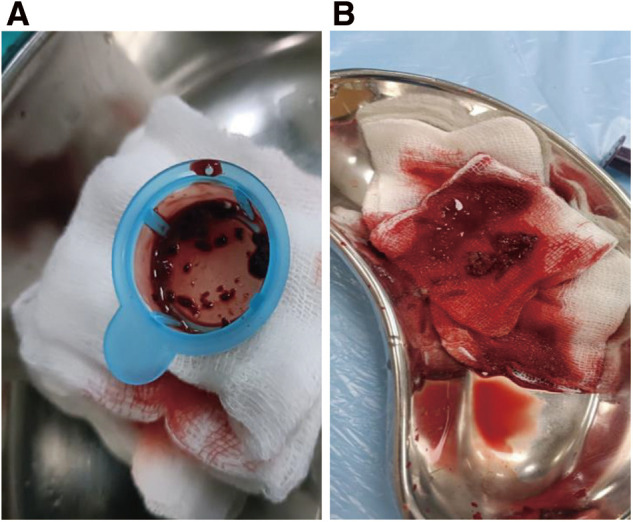
Fig. 2 (**A**) Thrombus aspirated from popliteal artery. (**B**) Aspirated thrombus.

At our institution, we conducted a study on 1020 TKA cases over 5 years from May 2017 to May 2022, with particular attention given to instances of acute limb ischemia (ALI) precipitated by popliteal artery thrombosis within the examined cohort. A detailed clinical assessment of each ALI case encompasses the palpation of pulses and a sensory-motor examination. Arterial Doppler ultrasound was performed on the affected limb, facilitating the evaluation of vessel integrity, blood circulation, and the degree and nature of obstruction within the lumen. The diagnosis of ALI was established by the identification of clinical manifestations such as pallor, paresthesias, paresis, and absence of pulses along with Doppler evidence indicating an occlusive event in the popliteal artery. Furthermore, the categorization of ALI severity was performed in accordance with the Rutherford classification system. All patients received endovascular treatment through the femoral artery opposite to the limb of surgery. Furthermore, we maintained regular follow-ups with these patients to monitor their management progress and prognosis. Thrombosuction was done with the guiding catheter itself followed by a thrombuster catheter and balloon inflations were done (**Figs. 1** and **2**). Thrombolytic agents and stents were not used. Four cases were treated within 7 hours of presentation, but 1 case was treated after 12 hours.

The steps of endovascular management followed in our cases are as follows: Initially, a peripheral angiogram of the lower limb was performed through the femoral artery approach. Once the thrombotic occlusion was identified, we passed a coronary angioplasty wire, keeping the catheter close to the occlusion. We aspirated the thrombus using a thrombuster catheter (typically used for coronaries) and also directly with the guiding catheter. Depending on the flow, we dilated the thrombotic lesion with a 3.5 × 18 mm or 4 × 23 mm coronary balloon, and for larger vessels, we used a 6 × 18 mm peripheral balloon over a Terumo (Tokyo, Japan) wire. Thrombus aspiration was repeated if needed. We did not use any intra-arterial thrombolytic agents and tried to minimize the use of contrast. All the steps of individual cases are detailed in **Table 1**. Though there is no direct therapy targeted at treating endothelial injury, balloon dilation and thrombus aspiration were performed to clear the thrombus. Aspirin, cilostazol, and statins will help in preventing further thrombus formation and treating endothelial dysfunction.

**Table:** Table 1 Clinical profile of all patients who underwent endovascular repair for popliteal artery thrombosis post-total knee arthroplasty

S No	Age/sex	Diagnosis	Risk factors	Tourniquet time	Timing of PTPA from the onset of symptoms	Angiogram	Procedure	Result	Follow-up
1	51/F	Left popliteal artery total occlusion	–	60 min	6 h	Thrombotic total occlusion of left popliteal artery with no flow distally	Right femoral artery approach. Thrombosuction and dilatation were done with a 4.0 x 23 mm balloon at 10 atm.	Final angio showed TIMI III flow in the popliteal artery, anterior and posterior tibial arteries, and dorsalis pedis artery	Foot drop recovered after 1 month. Color Doppler showed triphasic flow in all arteries.
2	60/M	Right popliteal artery total occlusion	–	80 min	7 h	Thrombotic total occlusion of right popliteal artery with no flow distally	Left femoral artery approach. Thrombosuction and dilatation were done with a 4.0 × 20 mm PTCA balloon at 20 atm and with a 6.0 x 18 mm peripheral balloon at 12 atm.	TIMI III flow in the popliteal artery and TIMI II flow in anterior, posterior tibial arteries, and dorsalis pedis artery	No complications. Arterial Doppler showed triphasic flow in all arteries of the left lower limb.
3	59/F	Left popliteal artery 99% occlusion	Diabetes mellitus, hypertension	75 min	6 h	99% occlusion of left popliteal artery with very faint flow distally	Right femoral artery approach. Thrombosuction and dilatation were done with a 3.5 x 28 mm PTCA balloon at 14 atm and 18 atm.	Good flow in the popliteal artery and TIMI II flow in anterior, posterior tibial arteries, and dorsalis pedis artery	No complications. Arterial Doppler showed triphasic flow in all arteries of the left lower limb.
4	60/F	Left popliteal artery tear with 100% thrombotic occlusion	Hypothyroidism	110 min	12 h	Left popliteal artery tear with thrombotic artery occlusion with no distal flow	Right femoral artery approach. Thrombosuction and dilatation were done in the left popliteal artery with a 3.5 x 38 mm balloon at 12 atm and 6.0 x 17 mm at 10 min.	TIMI III flow in Popliteal artery and TIMI II flow in anterior, posterior tibial arteries, and TIMI 1 flow in dorsalis pedis artery	Compartment syndrome, AKI, sepsis. Underwent dialysis. Foot drop persisted for 6 months.
5	54/M	Left popliteal artery tear with 100% thrombotic occlusion	Hypothyroidism	60 min	3 h	Mild thrombus in popliteal artery	Right femoral.	TIMI III in all arteries of the left lower limb	Normal arterial Doppler after 1 month.

PTPA: percutaneous transluminal peripheral angioplasty; PTCA: percutaneous transluminal coronary angioplasty; TIMI: thrombolysis in myocardial infarction; AKI: acute kidney injury

To ensure ethical standards, we obtained proper consent from the participants and approval from Alluri Sitarama Raju Academy of Medical Sciences—ASRAMS BHR ethics committee with approval number ASRAMS BHR-EC/Approval No. 09/2024.

The demographic features, clinical profiles, and treatment approaches of all patients are depicted in **Table 1**.

## Results

**Figure 3:**
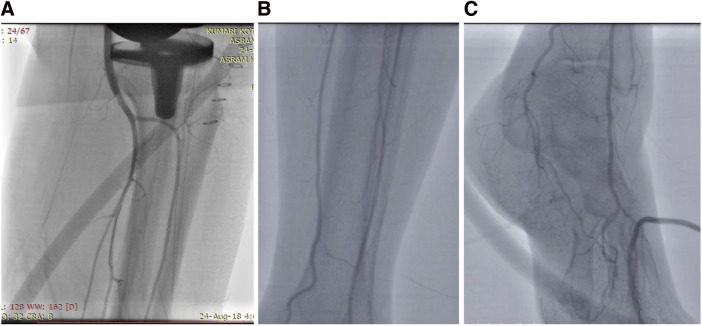
Fig. 3 (**A**) Images show the post-endovascular therapy outcome of the popliteal artery. (**B**) Anterior and posterior tibial artery demonstrating satisfactory blood flow. (**C**) Dorsalis pedis arteries demonstrate satisfactory blood flow.

**Figure 4:**
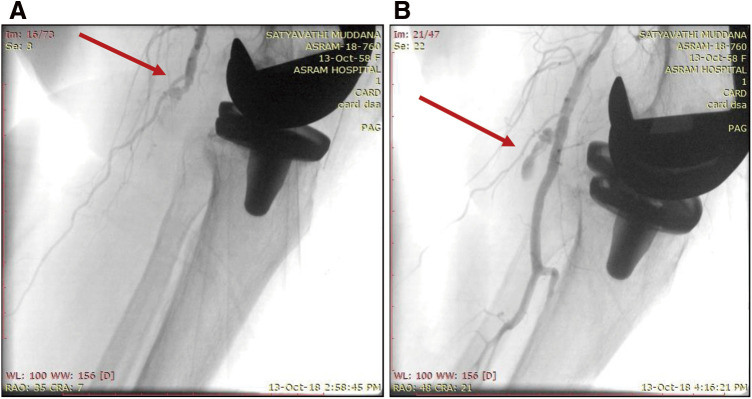
Fig. 4 (**A**) Aneurysm formation secondary to trauma to popliteal artery in left total knee arthroplasty with pre-angiographic image thrombotic occlusion of popliteal artery (red arrow). (**B**) Post-angiographic images after endovascular repair for thrombotic occlusion (red arrow).

Out of 1020 cases of TKA presented to our hospital during the study period, 5 cases of ALI were identified accounting for 0.49% of all TKA cases. Gender distribution revealed a slightly higher incidence in females, with a male-to-female ratio of 2:3. The age demographics were skewed toward middle-aged to older individuals ranging between 50 and 60 years of age. Notably, a significant predominance of left-sided presentations compared with right-sided lesions with a striking ratio of 4:1 (**Fig. 3**) was observed. Regarding comorbidities, 1 case each of diabetes mellitus, hypertension, and 2 cases with hypothyroidism was noted among the patients. None of our cases had atrial fibrillation before or during the course of their treatment. It is noteworthy that most patients sought medical attention promptly with presentations occurring within a timeframe of less than 12 hours from the onset of symptoms. Among the cases of ALI, 1 patient experienced a severe complication of compartment syndrome with sepsis, necessitating fasciotomy which subsequently led to renal failure requiring hemodialysis. Two patients presented with foot drop, indicating varying degrees of neurological involvement. Whereas another patient achieved recovery solely through anticoagulation therapy. One patient experienced a rupture of the left popliteal artery with aneurysm formation (**Fig. 4**), likely due to hyperextension, managed with prolonged balloon occlusion to seal the aneurysmal neck, thus underscoring the diverse treatment modalities and outcomes observed within the cohort. Four cases were under follow-up with no residual deformity after 3 years, while 1 case was lost to follow-up after the COVID-19 pandemic.

## Discussion

TKA stands out as the orthopedic surgical procedure most commonly associated with vascular injuries, followed by hip arthroplasty, spinal surgery, and knee arthroscopy. Specific risk factors linked to popliteal artery injury after TKA include revision surgery, peripheral vascular disease, renal failure, coagulopathy, and metastatic cancer.

### Incidence

Although there are numerous individual case reports of popliteal artery occlusion after TKA in elderly patients, only a few studies have conducted comprehensive analyses. Rand^4)^ reported a 0.03% occurrence rate among patients with TKA, whereas Calligaro et al.^5)^ reported 0.17%. Matziolis et al.^6)^ identified 31 cases of popliteal artery occlusion following TKA, resulting in 11 amputations. The incidence rate in our study was 0.49%, which is slightly higher than that reported in other significant studies. As ours is a referral tertiary care center, many cases have been referred for further management from various other centers. Hence, it reflects the incidence of multiple centers’ procedures, which has increased the overall incidence. Left-sided popliteal artery injuries were more prevalent in our case series; however, there was no specific explanation for the left-sided predominance.

### Etiopathogenesis

Virchow’s triad of damage to the arterial endothelium, intravascular stasis, and hypercoagulopathy suggests potential risk for the development of acute arterial thrombosis after TKA.^7)^ Various mechanisms, both direct and indirect, have been proposed to explain popliteal artery injury during TKA. Most popliteal vascular injuries result from direct trauma to the artery. Arterial laceration or transection is caused by a direct penetrating injury and may present as intraoperative hemorrhage, pseudoaneurysm, arteriovenous fistula, or recurrent hemarthrosis. Indirect mechanisms such as joint manipulation or tourniquet application can cause thrombotic occlusion and result in limb ischemia. During TKA, abnormal positioning of the knee is necessary to place the knee prosthesis adequately. Hyperextension may occur during patellar preparation, leading to significant tenting of the popliteal artery over the posterior joint line and putting it at risk.^8)^ The distance from the posterior capsule to the popliteal artery ranges approximately from 2.7 to 9.7 mm, varying with knee position and level, which increases the risk of injury to the artery. In addition, anterior displacement of the tibia during tibial cementing and large posterior osteophytes may pose significant perioperative risk factors for popliteal artery injury.

In revision TKAs, there is a higher risk of popliteal artery damage due to increased surgical correction needed for stiffened or distorted soft tissue, fixed flexion deformity, or varus/valgus deformity, potentially causing arterial kinking or transection. Although ischemic time due to tourniquet inflation is considered a risk factor for thrombosis, cases of arterial occlusion have been reported even with limited tourniquet use during the cementing phase.^9)^ Some authors recommend that in patients with ankle/brachial index <0.3 arthroplasty should be performed without the application of a tourniquet.^10)^ In only 1 case, the tourniquet duration exceeded 100 minutes, whereas in others, it was less than 80 minutes. Four of our cases who were treated within 7 hours recovered well without any significant complications.

### Complications

The complications associated with TKA include compartment syndrome, infection, neurological damage, amputation, and death, thus highlighting the importance of reperfusion within the first 6 hours post-injury.^11)^ In a report by Green and Allen, 86% of the patients required amputation if reperfusion of blood supply was done more than 8 hours after injury or occlusion of popliteal arterial blood flow due to trauma and dislocation of the knee.^12)^ Complications occurred in 2 of our cases, including compartment syndrome with sepsis and renal failure requiring dialysis and foot drop in 1 case and foot drop alone in another. The foot drop could be due to damage of the peroneal nerve because of ischemia and improved over 3–4 weeks, with physiotherapy and splint support with minimal residual deficit. These complications may have arisen because of reperfusion times exceeding 10 hours, especially for patients transferred from another hospital.^13)^ Pseudoaneurysm is another significant post-surgery complication. Multiple authors have reported delayed detection of popliteal artery pseudoaneurysm ranging from a few days to several months post-surgery.^14)^ Most of these patients initially exhibited no signs of vascular injury, with documented palpable distal pulses. One patient in our study population had a popliteal artery pseudoaneurysm and presented 36 hours postoperatively, which required prolonged balloon inflation to seal the neck of the aneurysm.

### Diagnosis

Although clinical examination is crucial for initial diagnosis, arterial Doppler study can aid in the diagnosis, and peripheral angiogram is typically more definitive for assessing the extent and associated complications of arterial occlusion.

### Treatment

**Figure 5:**
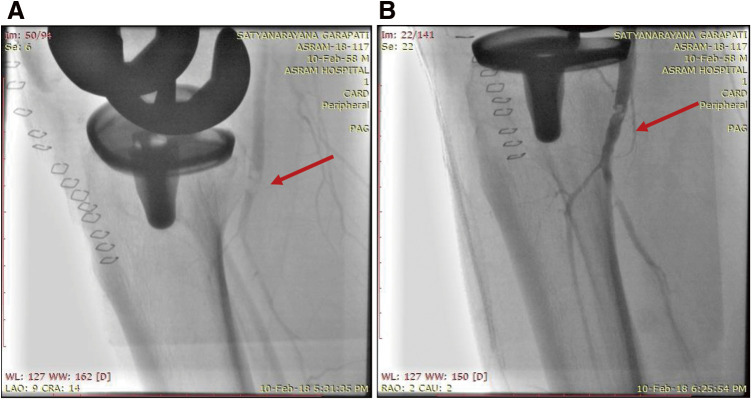
Fig. 5 (**A**) A pre-angiographic image red arrow shows thrombotic occlusion of the popliteal artery. (**B**) The post-angiographic image illustrates endovascular intervention for thrombotic occlusion in the left popliteal artery post-total knee arthroplasty with mild residual thrombus (red arrow).

In our study, we have preferred endovascular treatment over surgical management as it was more feasible, causes less morbidity, and offers faster recovery. Endovascular treatment (**Fig. 5**) is deemed safe, durable, and comparably effective to bypass grafting, particularly advantageous post-TKA because of its minimally invasive nature and proximity to the surgical site. Options include pharmacological or mechanical thrombolysis for thrombosis and angioplasty, stent application, or coil embolization for pseudoaneurysms.^15)^ Open surgery is necessary if endoscopic methods fail or for cases unsuitable for endoscopic treatment, like large vessel lacerations requiring repair, arterial tears, or pseudoaneurysms.^16)^ Surgical interventions include thrombectomy, vessel repair, excision followed by end-to-end anastomosis, arterial bypass, or above-the-knee amputation, with some patients requiring fasciotomy for delayed vascular repair. All cases except 1 underwent endovascular therapy, involving thrombosuction with a guiding catheter and balloon dilatation. Because this patient was a postoperative case, we avoided catheter directed thrombolysis (CDT) at the operative site, as it is a relative major contraindication. Although Supera stent^17)^ is a Food and Drug Administration (FDA)-approved stent for deployment in chronic diseases of the popliteal artery because of its very low risk of fracture, no stents were deployed because of the acute thrombotic nature of the lesions.

### Prevention

Preoperative assessment of radiographs to identify large posterior osteophytes aids in surgical planning. During surgery, it is essential to avoid excessive forces, particularly during tibial preparation and cementing to prevent kinking of the popliteal artery which can lead to endothelial injury. Strategies like avoiding tourniquet use, careful preoperative radiograph assessment, heightened perioperative vigilance, and patient optimization can further mitigate the already low risk of popliteal artery occlusion after TKA. Inserting retractors into the posterolateral corner of the tibia during TKA should be avoided because of the high vulnerability of the popliteal artery in this area.^18,19)^ Risk factors such as smoking, diabetes mellitus, hypertension, intermittent claudication, previous ischemic events, and clinical examination of lower extremity circulation are crucial. Ankle-brachial pressure index (ABPI) measurement serves as a simple preoperative bedside vascular study for atherosclerosis.^20)^ Palpating the dorsalis pedis artery post-TKA becomes vital for the early detection of ischemia, especially because typical signs may be obscured by anesthesia.^21)^

In our study, all cases underwent preoperative evaluation with a Doppler study but not with the ABPI. Postoperatively, follow-up was done with distal pulse examination and Doppler if needed. Now, we have made ABPI measurement mandatory for all cases pre- and post-TKA. The salient features of post-TKA popliteal artery thrombosis are summarized in **Table 2**.

**Table:** Table 2 Salient features of post-total knee replacement: popliteal artery thrombosis

1. Incidence	0.03%–0.17%
2. Etiopathogenesis	Endothelial injury by direct trauma intraoperative Posterior osteophytes or indirect injury by joint manipulation Tourniquet application Abnormal positioning like hyperextension of the knee Anterior displacement of the tibia during tibial cementing
3. Diagnosis	*Clinical*: High degree of suspicion in patients with purple discoloration of skin, pallor, pain, paresthesias, and absent distal pulse. *Radiological*: arterial Doppler, CT peripheral angiogram, or peripheral conventional angiogram.
4. Treatment	1. *Surgical interventions* include thrombectomy, vessel repair, arterial bypass, fasciotomy, and amputation if necessary. 2. *Endovascular interventions* include thrombectomy, pharmacological, or mechanical thrombolysis and angioplasty, and coil embolization for pseudoaneurysm.
5. Complication	Compartment syndrome Infection Neurological damage—peroneal nerve ischemia leading to foot drop
6. Prevention	*Preoperative:* By identifying large posterior osteophytes, clinical examination of distal pulses arterial pulses, and ankle-brachial pressure index along with arterial Doppler assessment. *Intraoperatively:* The surgeon should be cautious and avoid excessive forces during tibial preparation and cementing, careful tourniquet application, and avoid retractors into the posterolateral corner of the tibia.

## Conclusion

A high degree of suspicion and vigilance in the diagnosis and management of ALI secondary to popliteal artery thrombosis in cases of TKA is the emphasis of this study. Despite being a rare complication, the potential for significant morbidity and mortality underscores the need for prompt recognition and intervention. Endovascular treatment emerged as the preferred modality with effective management and reduced local complications. Preoperative vascular assessment, strict perioperative vigilance, and routine postoperative monitoring are vital to mitigate the risk of ALI. Continued research and implementation of evidence-based protocols are essential to further enhance patient safety and reduce the incidence of this potentially devastating complication.

## Declarations

### Informed consent

Informed consent was obtained from the patients.

### Funding

The authors have no funding disclosures.

### Disclosure statement

All authors have no conflict of interest.

### Author contributions

Study conception: TI, GKK, and VVS

Data collection: SNS, AK, and VVS

Analysis: TI and GKK

Investigation: TI, SNS, AK, and VVS

Manuscript preparation: TI, GKK, SNS, and VVS

Funding acquisition: not applicable

Critical review and revision: all authors

Final approval of the article: all authors

Accountability for all aspects of the work: all authors.

## References

[1] Turner NS 3rd, Pagnano MW, Sim FH. Total knee arthroplasty after ipsilateral peripheral arterial bypass graft: acute arterial occlusion is a risk with or without tourniquet use. J Arthroplasty 2001; 16: 317–21. 11307129 10.1054/arth.2001.21502

[2] Bayne CO, Bayne O, Peterson M, et al. Acute arterial thrombosis after bilateral total knee arthroplasty. J Arthroplasty 2008; 23: 1239.e1–6. 10.1016/j.arth.2007.11.012 18534431

[3] Ogawa H, Matsumoto K, Ito Y, et al. Indirect popliteal artery transections in revision total knee arthroplasty: a case report. Bull Hosp Jt Dis 2016; 74: 168–71. 27281324

[4] Rand JA. Vascular complications of total knee arthroplasty. J Arthroplasty 1987; 2: 89–93. 3612144 10.1016/s0883-5403(87)80014-1

[5] Calligaro KD, DeLaurentis DA, Booth RE, et al. Acute arterial thrombosis associated with total knee arthroplasty. J Vasc Surg 1994; 20: 927–32; discussion, 930-2. 7990187 10.1016/0741-5214(94)90229-1

[6] Matziolis G, Perka C, Labs K. Acute arterial occlusion after total knee arthroplasty. Arch Orthop Trauma Surg 2004; 124: 134–6. 14658074 10.1007/s00402-003-0602-0

[7] Tsujimoto R, Matsumoto T, Takayama K, et al. Acute popliteal artery occlusion after revision total knee arthroplasty. Case Rep Orthop 2015; 2015: 672164. 26357582 10.1155/2015/672164 PMC4556868

[8] Ninomiya JT, Dean JC, Goldberg VM. Injury to the popliteal artery and its anatomic location in total knee arthroplasty. J Arthroplasty 1999; 14: 803–9. 10537254 10.1016/s0883-5403(99)90029-3

[9] Fukuda A, Hasegawa M, Kato K, et al. Effect of tourniquet application on deep vein thrombosis after total knee arthroplasty. Arch Orthop Trauma Surg 2007; 127: 671–5. 17102960 10.1007/s00402-006-0244-0

[10] Langkamer VG. Local vascular complications after knee replacement: a review with Illustrative Case Reports. Knee 2001; 8: 259–64. 11706687 10.1016/s0968-0160(01)00103-x

[11] Imanaka T, Fujii K, Fukunaga M, et al. A case of acute thrombotic occlusion of the popliteal artery occurring immediately after the total knee arthroplasty recanalized by ballooning alone. J Cardiol Cases 2013; 8: 190–2. 30534289 10.1016/j.jccase.2013.08.008 PMC6277696

[12] Green N, Allen B. Vascular injuries associated with dislocation of the knee. J Bone Joint Surg Am 1977; 59: 236–9. 845209

[13] Defraigne JO, Pincemail J. Local and systemic consequences of severe ischemia and reperfusion of the skeletal muscle. Physiopathology and prevention. Acta Chir Belg 1998; 98: 176–86. 9779243

[14] Hozack WJ, Cole PA, Gardner R, et al. Popliteal aneurysm after total knee arthroplasty. Case reports and review of the literature. J Arthroplasty 1990; 5: 301–5. 2290084 10.1016/s0883-5403(08)80087-3

[15] Troutman DA, Dougherty MJ, Spivack AI, et al. Updated strategies to treat acute arterial complications associated with total knee and hip arthroplasty. J Vasc Surg 2013; 58: 1037–42. 23747133 10.1016/j.jvs.2013.04.035

[16] Feliciano DV, Moore FA, Moore EE, et al. Evaluation and management of peripheral vascular injury. Part 1. Western Trauma Association/critical decisions in trauma. J Trauma 2011; 70: 1551–6. 21817992 10.1097/TA.0b013e31821b5bdd

[17] Werner M, Paetzold A, Banning-Eichenseer U, et al. Treatment of complex atherosclerotic femoropopliteal artery disease with a self-expanding interwoven nitinol stent: midterm results from the Leipzig SUPERA 500 registry. EuroIntervention 2014; 10: 861–8. 24682531 10.4244/EIJV10I7A147

[18] Karanam LS, Busireddy NR, Baddam SR, et al. Acute thrombotic occlusion after total knee arthroplasty: role of endovascular management. J Clin Orthop Trauma 2018; 9: 121–4. 29896013 10.1016/j.jcot.2016.12.010 PMC5995678

[19] Farrington WJ, Charnley GJ. The effect of knee flexion on the popliteal artery and its surgical significance. J Bone Joint Surg Br 2003; 85: 1208–1208; author reply, 1208. 14653611

[20] Parvizi J, Pulido L, Slenker N, et al. Vascular injuries after total joint arthroplasty. J Arthroplasty 2008; 23: 1115–21. 18676115 10.1016/j.arth.2008.02.016

[21] Raju IT. Acute limb ischemia secondary to popliteal artery thrombosis following total knee arthroplasty – limb salvage by endovascular therapy. Indian J Vasc Endovasc Surg 2018; 5: 115–118.

